# A Systematic Review of a Polyvagal Perspective on Embodied Contemplative Practices as Promoters of Cardiorespiratory Coupling and Traumatic Stress Recovery for PTSD and OCD: Research Methodologies and State of the Art

**DOI:** 10.3390/ijerph182211778

**Published:** 2021-11-10

**Authors:** Andrea Poli, Angelo Gemignani, Federico Soldani, Mario Miccoli

**Affiliations:** 1 Department of Clinical and Experimental Medicine, University of Pisa, 56126 Pisa, Italy; mario.miccoli2020@virgilio.it; 2 Department of Surgical, Medical and Molecular Pathology and of Critical Care Medicine, University of Pisa, 56126 Pisa, Italy; gemignan@dfb.unipi.it; 3 U.K. National Health Service, Aberdeen AB25 2ZH, UK; federico.soldani@doctors.net.uk

**Keywords:** polyvagal theory, contemplative practices, mindfulness, traumatic stress, study designs, data analysis methods

## Abstract

Baseline respiratory sinus arrhythmia (RSA) has been proposed as a transdiagnostic biomarker of stress vulnerability across psychopathologies, and a reliable association between PTSD, OCD and lower resting RSA was found. Contemplative practices have been linked to the activation of the vagus as well as to an increased RSA that, according to the polyvagal theory, reflects the activation of the ventral vagal complex (VVC) and may promote PTSD and OCD recovery. PubMed and Scopus databases were selected to conduct a search following the Preferred Reporting Items for Systematic Review and Meta-Analyses (PRISMA) 2020 guidelines, and A MeaSurement Tool to Assess systematic Reviews-2 (AMSTAR-2) was used to appraise the methodological quality for this systematic review. Six articles met the inclusion criteria (one cross-sectional study, one study with pre-post measurements, two cohort studies and two RCT studies). Mindfulness-related interventions promoted parasympathetic activity, an increased vagal tone and improvements in PTSD and OCD symptoms. According to the polyvagal theory, mindfulness-related and compassion-related meditations would be conceptualized as neural exercises expanding the capacity of the ventral vagal complex to regulate the present state and to promote resilience. Clinical and methodological issues are discussed.

## 1. Introduction

### 1.1. Rationale

The polyvagal theory [1,2,3] proposes an evolutionary and neurophysiological framework that deals with the organization of autonomic systems and has been linked to functional gastrointestinal disorders and chronic diffuse pain [4] and to psychosocial pathology [5], both connected to chronic traumatic stress during development. Considering the evidence derived from neurophysiology, behavioral observations and comparative anatomy, the polyvagal theory proposes the existence of two separate vagal pathways belonging to the parasympathetic nervous system that give rise to the emergence of a dorsal vagal complex (DVC) and a ventral vagal complex (VVC).

In accordance with the polyvagal theory [1,2], the function and the structural organization of the autonomic nervous system of the human being are embedded in its phylogenetic origin through a hierarchical organization. The social engagement system (SES) originates from the myelinated VVC, whose fibers are cardioinhibitory and stem from the brainstem, in particular in the nucleus ambiguus (NA). The VVC is a threat-response system that tends to preserve homeostasis, is the most recent at the phylogenetic level and tends to act rapidly (considering its myelinated fibers). The sympathetic nervous system (SNS) is less recent than the VVC; its activation favors the frequency increase in heart rate, respiration, and mobilization for active threat responses such as escape or fight defense. The dorsal vagal complex (DVC) shows unmyelinated cardioinhibitory fibers that are rooted in the dorsal motor nucleus of the vagus (DMNX) of the brainstem. Phylogenetically, it is the less recent of the autonomic subsystems and presents a vestigial immobilization function that emerged in early vertebrates. Threat reactions and homeostasis are related to the DVC, which innervates organs below the diaphragm as well. The DVC is also responsible for the disruption of digestive mechanisms and the preservation of metabolic energies when it is recruited during reactions to threats [1,2]. Remarkably, the DVC represents the system that is mainly recruited in traumatic responses after psychological trauma [4,5].

The polyvagal theory proposes that the integration of the myelinated cardiac vagal pathways with the neural regulation of the head and face favored the advent of the SES of mammalians. The outputs of the SES include motor pathways that control striated muscles of the head and face (i.e., somatomotor) and cardiac and smooth muscles of the bronchi and heart (i.e., visceromotor). The somatomotor portion includes special visceral efferent pathways that controls the striated muscles of the head and face. The visceromotor portion includes the myelinated supradiaphragmatic vagal pathway that controls the bronchi and heart. At the functional level, the SES arises from a face-heart connection that harmonizes the heart with the muscles of the head and face. The inceptive function of the system is to orchestrate swallowing, sucking, vocalizing and breathing. A disruption of the SES during early phases of life is an index of later problems in social skills and emotional homeostasis. The neural detection of environmental risk establishes the preferential activation of the SES, or the subsequent hierarchical progressive recruitment of the SNS or the DVC.

In accordance with the polyvagal theory, neuroception is a neural process different from perception that controls the neural detection of risk and does not need a conscious awareness. In addition, neuroception is carried out through a neural reflexive process that is able to immediately shift physiological state and to distinguish visceral and environmental characteristics that are safe, dangerous, or life-threatening. When environments are safe, a neuroception of safety favors the SES, and the SNS activation is adaptively dampened to preserve the central nervous system that is highly oxygen-dependent, in particular the cortex, from the activities of the DVC (e.g., fainting) that are highly metabolically conservative. In contrast, a neuroception of danger, or life threat, favors activation of the SNS or DVC, respectively [1,2,6]. The organization of these specific circuits, along with the SNS, can influence the individual experiences of body awareness by modulating information that emerges from the body through top-down post-processing, including cortical areas informed by the signals traveling through the body-integrative circuits of the brain [7,8].

The World Health Organization (WHO) defined health as a “state of complete physical, mental and social well-being and not merely the absence of disease or infirmity” [9]. Health implies rhythmicity and timing inside and among individuals [10]. Thus, health implies a state of interconnectedness [11]. Interestingly, experiencing interconnectedness and compassion has been linked to the activation of the vagus [12] as well as to an increased respiratory sinus arrhythmia (RSA) [13] that, according to the polyvagal theory, reflects the activation of the VVC. 

Contemplative practices, characterized by the attentive regulation of breathing, are believed to be mediated by vagal activation [14,15,16], and an increasing number of contemplative practices are being used as “health” interventions for individuals with different health conditions. Recent research has shown that contemplative practices are successful at ameliorating many different morbid situations that encompass cardiovascular disease [17], post-traumatic stress disorder (PTSD) [18], vascular disease [17], fibromyalgia [19], lower back pain [20,21,22,23,24], hypertension [25], and obsessive-compulsive disorder (OCD) [26,27,28,29,30,31,32]. In accordance with these observations, a recent meta-analysis investigating baseline RSA as a psychophysiological marker of stress vulnerability in individuals with PTSD in 55 studies revealed a small but reliable association between PTSD and lower resting RSA [33], as well as the display of lower RSA in OCD patients compared to healthy controls [33,34]. Thus, contemplative practices may be beneficial at the individual as well as the population level, and RSA has been proposed as a transdiagnostic biomarker of emotion dysregulation [35].

Common forms of contemplative practices include mindfulness, compassion and self-compassion, and Tai Chi/Qigong or yoga often practiced 20 min or more, once or twice daily, but can extend into daily life as well [11]. However, mindfulness-related and compassion-related practices are among the most common forms of meditation [36], and three kinds of meditation are considered among “mindfulness meditations” in the West: focused attention, open monitoring (attentional practices), and loving-kindness and compassion (constructive practices) [37,38]. Focused attention requires bringing the attention back, every time that is needed, to the breath or to a specific object; open monitoring (also called choiceless awareness) involves noticing what is most prominent and important, moment-to-moment, in the domain of awareness; loving-kindness meditation is related to an intentional cultivation of happiness while compassion meditation involves cultivating goodwill in the face of suffering [38]. While focused attention and open monitoring involve focusing on a specific object or on a part of the self, such as cognition, emotion, or perception, loving-kindness and compassion meditation require the self as the focus of practice [38,39,40].

In particular, Paul Gilbert and Choden, a Buddhist monk, define compassion as a sensitivity to the suffering of the self and the others with a commitment to try to alleviate and prevent it [41], while Kristin Neff defines self-compassion as ‘being open to and moved by one’s own suffering, experiencing feelings of caring and kindness toward oneself’ [42]. Brain imaging studies confirm the idea of considering compassion as more involving, at the emotional level, than mindfulness. Practicing compassion promotes the activation of regions of the positive affect system, such as the nucleus accumbens, the ventral striatum and the medial orbitofrontal cortex [43,44,45]. In this review, we will mainly focus on the effects of mindfulness-related and compassion-related contemplative practices on traumatic stress conditions, such as PTSD and OCD (which has been associated with a significant overlap of traumatic histories) [46], from a polyvagal perspective.

### 1.2. Objectives

The aim of this systematic review was to identify common psychophysiological mechanisms underlying the beneficial effects of mindfulness and compassion contemplative practices by systematically reviewing studies in the scientific literature and their research methodologies.

## 2. Materials and Methods

### 2.1. PICOS

Only studies related to traumatic stress conditions, such as PTSD or OCD [46], and involving humans were included. We did not include research that relied on self-reporting instruments only, since the reliability of this type of research is questioned by the absence of factual measures, one of the main problems related to the contemplative sciences [47]. Our review concentrated on research studying modifications of physiological indexes relevant to central and/or autonomic nervous system activity, and their associations with behavior.

In our systematic review, we included physiological parameters such as autonomic activity, investigated through respiratory sinus arrhythmia (RSA), cardio-respiratory synchronization and heart rate variability (HRV), and brain activity, investigated through fMRI and electroencephalography (EEG).

We adopted the Population, Intervention, Comparison, Outcomes and Study (PICOS) design strategy (see Methods and Table 1), in order to carry out an effective search strategy.

### 2.2. Search Strategy

This systematic review followed the Preferred Reporting Items for Systematic Reviews and Meta-Analyses (PRISMA) guidelines [48,49] and recent PRISMA 2020 updates [50]. PRISMA is a 27-item tool that serves as a checklist to improve the quality of systematic reviews [48,49]. We submitted the protocol for this review for pre-registration in the PROSPERO database, an international prospective register for systematic reviews. It has been assigned the ID number 268353 (https://www.crd.york.ac.uk/prospero/, accessed on 1 November 2021).

Using SCOPUS and PubMed databases, we conducted a systematic search: the first search was conducted in April 2021, and the final search was carried out in July 2021. The Boolean operators “AND”, “OR” and “()” were used in order to combine keywords related to mindfulness, compassion, traumatic stress, OCD, and PTSD and to the physiological outcomes of mindfulness-related and compassion-related interventions in OCD and PTSD. The search for the mindfulness-related and compassion-related interventions used the combination of the following keywords: “mindfulness” OR “MBSR” OR “compassion” OR “self-compassion”. The search for the physiological effects used the association of the following keywords: “Cardiorespiratory coherence” OR “Cardio respiratory coherence” OR “Cardio-respiratory coherence” OR “Cardiorespiratory coupling” OR “Cardio respiratory coupling” OR “Cardio-respiratory coupling” OR “Cardiorespiratory interaction” OR “Cardio respiratory interaction” OR “Cardio-respiratory interaction” OR “Cardiorespiratory synchronization” OR “Cardio respiratory synchronization” OR “Cardio-respiratory synchronization” OR “Electroencephalogram” OR “EEG” OR “Functional connectivity” OR “Heart rate variability” OR “HRV” OR “Magnetic resonance imaging” OR “MRI” OR “Respiratory Sinus Arrhythmia” OR “RSA”. Combining the keywords “Post-traumatic stress disorder” OR “PTSD” OR “Obsessive-compulsive disorder” OR “OCD” was carried out as a search for PTSD and OCD conditions. We conducted the search both for acronyms and extended names.

### 2.3. Study Design

We established the inclusion criteria and the exclusion criteria for our review, following the PICOS strategy (Table 1). Studies found during the literature search were considered in the review if all of the following criteria were met:

- They were carried out on humans diagnosed with PTSD or OCD (whether they were expert or naïve about mindfulness or compassion techniques).
- Any protocol of mindfulness-related or compassion-related intervention was used.
- Mindfulness-related or compassion-related interventions compared with control groups (other interventions, no intervention) were included.
- Physiological parameters associated with the central nervous system or cardio-respiratory system (i.e., cardio-respiratory synchronization, RSA, fMRI, EEG, HRV) and measured through a behavioral/psychological variable (investigated through a psychometric quantitative tool).

Studies found during the literature search were not considered in the review if:

- Underaged (under 18 years) and/or older (over 65 years) subjects.
- Slow breathing procedures not related to mindfulness or compassion interventions.
- Physiological parameters that were not relevant for the review, or only a behavioral/psychological index alone was investigated.
- They considered case reports.
- A lack of rigor in the description of the methodological process (e.g., age, gender, how subjects were recruited, treatments and procedures used that are clearly stated, protocol duration, measured outcomes) and of the experimental tools (e.g., tools and setups used to measure outcomes), such that replicability is difficult.
- They were not published in a peer-reviewed journal.
- They were not available in English language and/or in full-text.

The AMSTAR-2 (A MeaSurement Tool to Assess systematic Reviews-2) [51] approach was used to appraise the methodological quality of our review. A more specific evaluation of systematic reviews that comprises randomized or non-randomized studies of mindfulness- or compassion-related interventions, or both, is possible when using AMSTAR-2. Two independent raters (AP and MM) evaluated each study, and, when discrepancies were present, divergences were resolved through a meeting, referring to a third rater (AG), if necessary, until agreement was achieved.

## 3. Results

### 3.1. Search Results

**Figure 1:**
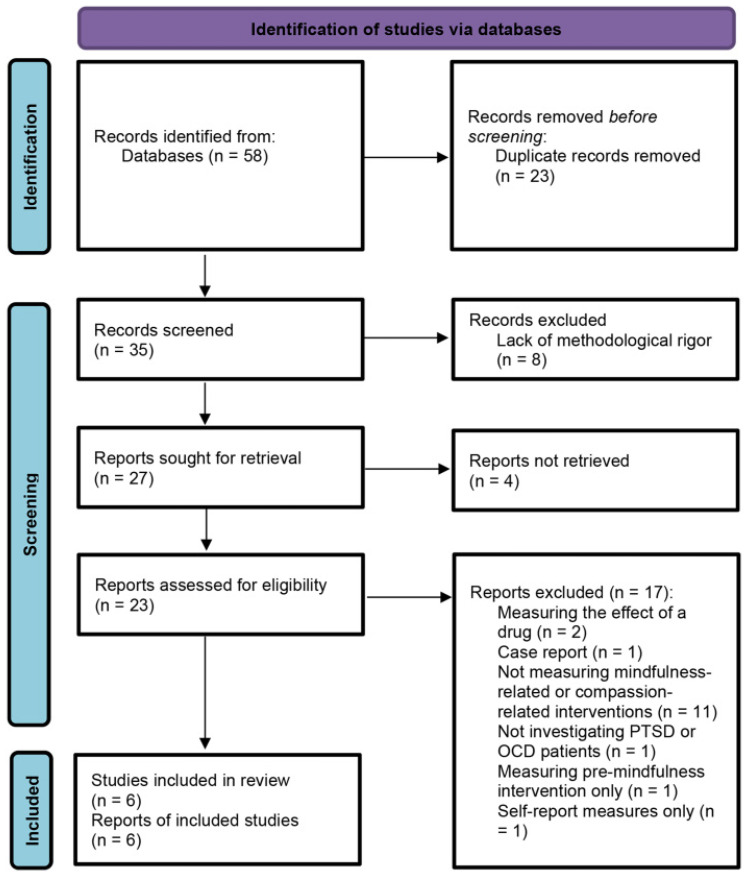
PRISMA 2020 flow chart for literature search and screening results.

The results of the search of databases, according to the chosen queries and quantity of studies found, are presented in Table 2. The PRISMA 2020 flowchart related to the process of selecting studies that were considered in the review is shown in Figure 1.

### 3.2. Selection and Characteristics of the Considered Studies

AP and MM were the two separate reviewers that checked an early pool of 58 abstracts gathered from the outcome of the search. Abstracts and titles were reviewed, and 35 studies were discarded because they were not retrieved, duplicated, or of no interest for our systematic review. A total of 23 remaining full-text papers were evaluated for the eligibility criteria. After the analysis, six articles met the eligibility criteria and were considered in the review. One study [52] was cross-sectional and dealt with the investigation of the effects of attentional distraction in 21 unmedicated OCD patients. One study was a pre-post study [53] and examined potential neural correlates of mindfulness-based exposure therapy (MBET). Two studies were cohort studies [54,55] and investigated the effects of MBSR intervention on PTSD symptoms and HRV in combat veterans [54], and the effects of transcendental meditation (TM) on 29 veterans with PTSD using EEG recordings [55]. One study was an RCT study [56] that aimed to evaluate the effect of two common components of meditation (mindfulness and slow breathing) investigating HR, HRV, awakening cortisol and PTSD measures collected through the CAPS, while Williams et al. [57] describe the protocol of an RCT to evaluate the effectiveness and mechanisms of three manualized 8-week behavioral interventions, MM, hypnosis (HYP) and education control (EDU), in 343 veterans with PTSD and chronic pain due to a broad range of etiologies.

We describe the methodologies used in the considered studies and their most important results in Table 3 and Table 4, respectively.

### 3.3. Synthesized Findings

#### 3.3.1. Cross-Sectional Studies

Simon et al. [52] investigated the effects of attentional distraction [58] in 21 unmedicated OCD patients, with respect to 21 controls, while undergoing fMRI during symptom provocation with individually tailored OCD-relevant pictures. Fronto-striato-limbic circuits included the amygdala, which is relevant in OCD [59,60]. Patients showed increased blood oxygenation level-dependent (BOLD) responses during processing of OCD triggers compared with healthy controls. This result shows that amygdala hyperactivation was present across every OCD symptom dimension, indicating that it may represent a common neural correlate. Attentional distraction was able to dampen patients’ amygdala hyperactivity: BOLD response of the left amygdala in relation to OCD triggers was halved only during distraction condition (*p* = 0.001), ameliorating OCD symptomatology. Overall, these results show that amygdala hyperactivation might constitute a shared correlate of fear expression in OCD dimensions, linking the OCD spectrum to anxiety disorders.

#### 3.3.2. Pre-Post Studies

King et al. [53] examined potential neural correlates of mindfulness-based exposure therapy (MBET), investigating 23 veterans with PTSD. Fourteen combat veterans with PTSD were treated with MBET, while 9 combat veterans, as a control group, were treated with present-centered group therapy (PCGT). Potential neural correlates were evaluated using fMRI and examining resting-state functional connectivity (rs-FC) in the default mode network (DMN), and PTSD symptoms were evaluated with the CAPS. Results showed that patients treated with MBET showed the greatest reduction in PTSD symptoms (pre vs. post MBET, *p* = 0.007, average 15.6 point decrease in total CAPS, effect size Cohen’s d = 0.92), even though the effect was not significantly different from PCGT. Following MBET, an increased DMN rsFC with DLPFC and dorsal ACC regions associated with executive control was shown. Eventually, posterior cingulate cortex-DLPFC connectivity was correlated with improvement in PTSD avoidant and hyper-arousal symptoms.

#### 3.3.3. Cohort Studies

Bhatnagar et al. [54] investigated the effects of an 8-week MBSR intervention on eight veterans with PTSD (seven men from the Vietnam era and one woman from Operation Enduring Freedom/Operating Iraqi Freedom). The main outcome measures were the Clinician Administered PTSD Scale (CAPS) [61,62] and the pNN50 measure of HRV (the number of pairs of adjacent normal-to-normal (NN) intervals differing by more than 50 milliseconds divided by the total number of all NN intervals). These were collected by interview and 24-h Holter monitoring at baseline (week 0), upon completion of the course (week 8), and 1 month after completion (week 12). One month after course completion, PTSD symptoms had decreased from baseline by an overall CAPS score of 14.8 points; this reduction was clinically significant according to prior studies [63]. Parasympathetic functioning increased for all five participants that underwent HRV monitoring.

Transcendental meditation (TM) represents a mental strategy that uses a mantra to promote meditation. Kang et al. [55] investigated 29 veterans with PTSD (six females) recruited from a medical center and included in the study. TM instructions were given by certified TM teachers trained at the Maharishi Foundation. TM provided 8 weeks of individual and group-based meditation practice. Outcomes of the study consisted of clinical interviews, self- report tools, and EEG recorded during resting and meditation conditions. Participants showed reductions in PTSD symptoms, experiential avoidance, and depressive and somatic symptoms, as well as increases on scores of mindfulness and quality of life, from baseline to posttreatment. Overall treatment improvement was high (mean and median rating of a 9-point scale were 6.2 and 7). With respect to baseline, low-frequency bands (1–7 Hz) of EEG spectral power increased at posttreatment and follow-up and only when meditation states were present (*p* = 0.01), suggesting TM-related modifications in brain state associated with the intervention.

#### 3.3.4. Randomized Controlled Trials (RCTs)

Wahbeh et al. [56] sought to assess the effects of two well-known parts of meditation (mindfulness and slow breathing). The authors studies 102 combat veterans suffering from PTSD that were randomized to four conditions: (a) the body scan [64] mindfulness meditation (MM), (b) slow breathing (SB) with a biofeedback device, (c) MM with an intention to slow the breath (MM + SB), or (d) sitting quietly (SQ). Participants underwent six weekly one-on-one sessions followed by 20 min of daily practice at home. The most important measures obtained were HR, HRV and cortisol after awakening. PTSD scores were assessed using the CAPS. Results demonstrated that the awakening cortisol was reduced within the MM group, but there was no overt change in HR and HRV within and between groups. However, there was an improvement in relation to subjective hyperarousal symptoms within-group (but not between groups) for MM, MM plus SB, and SQ, while intrusive thoughts were reduced in MM compared with MM plus SB and SB alone. Overall, mindfulness changes, comparing before and after intervention, were significant (*p* = 0.04).

Williams et al. [57] describe the protocol of an RCT to assess the efficacy and the functioning mechanisms of three manualized 8-week interventions: MM, hypnosis (HYP) and education control (EDU) in 343 combat veterans suffering from PTSD and chronic pain stemming from various different etiologies. The main goal of the study was to compare the efficacy of MM and HYP to EDU on average pain intensity assessed pre- and post-treatment. In addition, the study aimed to explore the efficacy of MM and HYP compared to EDU on some secondary results (i.e., sleep quality, pain interference, anxiety and depression), and the maintenance of its effects at 3 and 6 months post-treatment. EEG assessments were carried out at pre- and post-treatment to establish if the power of specific brain oscillations was able to moderate the efficacy of MM and HYP and evaluate brain oscillations as possible mediators of the effects of the treatment.

#### 3.3.5. Risk of Bias

The methodological quality and the risk of bias of the considered studies were independently evaluated by AP and MM, using four different tools. A third reviewer (AG) resolved possible discrepancies through a discussion with the reviewers (AP and MM). The Joanna Briggs Institute (JBI) critical appraisal checklist for cross-sectional studies, pre-post studies, cohort studies and RCTs [65,66] was used to appraise the risk of bias of cross-sectional, pre-post, cohort and RCTs studies, respectively. The four tools that were used for the assessment showed study quality that was considered varied from sufficient to good. Normality tests were lacking (e.g., Shapiro–Wilk test). Regarding cross-sectional study design, the most important concerns were related to the identification of confounding factors identified and measurement of the exposure in a valid and reliable way. When considering pre-post and cohort studies, the main concerns were related to the methods of sampling, sample sizes that may be statistically unjustified, and the lack of randomization for group assignment. Regarding cross-sectional, pre-post and cohort studies, power analysis was not reported and sample sizes were not appropriately identified. Checklists that were related to the risk of bias are shown for cross-sectional, pre-post, cohort and RCT designs in Supplementary Tables S1–S4, respectively.

## 4. Discussion

Conducting a revision of the studies that pertained to the psychophysiological outcomes of mindfulness-related and compassion-related interventions on traumatic stress conditions, such as PTSD and OCD, we aimed at identifying the physiological mechanisms that may be able to mediate their demonstrated beneficial psychological and behavioral efficacy. We found a paucity of evidence that highlighted a link between physiological variables and psychological and/or behavioral measures in PTSD and OCD patients.

Within PTSD patients, several results suggest that mindfulness-related interventions promote parasympathetic activity, an increased vagal tone and PTSD symptom improvements. The increased pNN50 measure of HRV [54], the increased DMN rsFC with DLPFC and dorsal ACC regions following MBET [53] and the increased low-frequency bands (1–7 Hz) of EEG spectral power, at post-treatment and follow-up, and only during meditation states [55] are results that favor the parasympathetic activity-promoting interpretation. Remarkably, though no change was observed in HR and HRV within and between groups by Wahbeh et al. [56], Wahbeh et al. [56] did not use an MBSR protocol, whereas it was used by Bhatnagar et al. [54]. In addition, an observed dampening of BOLD activity in the amygdala was reported by Simon et al. [52] in OCD patients. According to the polyvagal theory, mindfulness-related and compassion-related meditations would represent neural exercises broadening the ability of the VVC to govern the current state and to favor resilience. In addition, mindfulness-related and compassion-related meditations comprise a range of practices whose aim is to accomplish a similar efficacy in improving autonomic regulation, providing greater psychological and physiological flexibility and tolerance by leveraging emotional reactivity and ameliorating the physiological reactivity threshold [67].

Hence, mindfulness-related and compassion-related meditations are believed to favor the experience of a neural training, and a way to favor the activity of the VVC whose aims are the regulation, homeostasis and resilience of the organism [12]. Through the modification of the relational awareness towards the physiological state of the neural platforms proposed by the PVT, the physiological reactivity threshold can be strengthened [68]. Mindfulness-related and compassion-related meditations may favor the availability of the VVC to support the mechanisms of physiological recovery and positive behavioral and psychological states. Mindfulness-related and compassion-related meditation practices may also be viewed as promoters of the development of the flexibility as well as of the readiness to shift in and out of the current dominance of one of these theoretical neural platforms (DVC, SNS and VVC), in order to cultivate resilience and evolutionary adaptation of the system. Learning strategies for self-regulation may help individuals to improve their relationship to suffering.

Although we are not proposing that the only final goal is the cultivation of VVC, the theoretical association with the neural substrate of the VVC may be viewed as a neurophysiological platform for the advent of states such as social connection. Analogously, we do not wish to propose that the neural platforms other than VVC are “bad”, as these neurophysiological states are adaptive in comprehending the complexity of human behavior and experience, and thus the potential influence of a mindfulness-related and compassion-related meditation framework for well-being.

Thus, since mindfulness-related interventions promote parasympathetic activity, an increased vagal tone and symptom improvements, it can be hypothesized that many of the processes associated with contemplative practices (e.g., listening, chanting, breathing, shifting posture, and facial expressivity) may influence one’s physiological state through the myelinated branch of the vagus (VVC). The passive pathway recruits the SES (including the myelinated ventral vagus) through the cues of safety, such as a quiet environment and the presentation of prosodic vocalizations (e.g., chants) in the frequency band that would overlap with the vocal signals of safety that a mother uses to signal safety to her infant [69]. Hence, mindfulness-related and compassion-related interventions optimize the effects of contemplative training, leading to a greater capacity to feel and express presence and compassion. The factors involved may be: (1) a “passive” pathway that is elicited by feeling safe in an environment that provides sensory cues that, via neuroception, down-regulate defense; (2) an “active” pathway that is implemented via voluntary behaviors (i.e., neural exercises of the vagal brake) capable of establishing a “calm” neural platform (i.e., ventral vagal state) that would functionally optimize contemplative practices; (3) extensive contemplative training; and (4) the emergent properties of contemplative practices, including the capacity to experience and express compassion [69].

From the methodological point of view and statistical analyses, several clinical research studies employed small sample sizes, which in turn lowered statistical power. A recent review reported that only 0.3% of the included studies had ≥80% power to detect a small difference and from 22% to 27% of the studies had power to detect a large difference [70]. In addition, among the studies that did not detect a statistically significant effect, 3% excluded a small difference, and 71% excluded a large difference. Hence, an important pool of clinical research studies was powered only to detect effects of large magnitude. For most studies that do not reach statistical significance, the possibility of relevant differences still exists. However, it could be highly significant for research regarding contemplative practices to evaluate and include power analysis and appropriate sample size calculations, since studies point to beneficial effects of meditation and mindfulness on cognition, affect, and social behavior [71,72,73] (for review see Dahl et al. [40]; Tang et al. [74]), but their effect often may be identified within a narrow window of small effect sizes that require appropriate samples to be detected [75]. In addition, a small effect size could correspond to a highly significant finding, depending on the sample size [76].

Limitations of our systematic review should be considered. Our study included only Scopus and PubMed databases for the identification of potentially eligible studies; additional databases should be considered in future studies. The small number of studies is another limitation of this review, and the present results should be considered preliminary; future reviews may include additional studies and confirm, or revise, our findings. Another limitation concerns the fact that only one study included in the review assessed the effects of TM at follow-up, and this prevented the evaluation of the effects long-term. Furthermore, four out of six studies did not report power analysis, raising the possibility of underpowered study designs. Finally, pharmacotherapy for psychiatric disorders has been shown to have an influence on HRV [77]. In fact, treatment of major depression (selective serotonin reuptake inhibitors (SSRIs) and serotonin and norepinephrine reuptake inhibitors (SNRIs)) was able to influence autonomic function far more than the disease itself [78]. In addition, though SSRIs have been shown to have less impact on HRV than other antidepressants, results suggest that reductions in HRV observed among depressed older adults are driven by the effects of antidepressant medications [79]. Thus, future research regarding contemplative practices and HRV monitoring should control for the effects of psychoactive medications.

Despite these limitations, our review has several strengths. We provided a detailed search strategy for literature search and study identification, and appraised the methodological quality of this systematic review and of the included studies. In addition, we considered methodological aspects such as power analysis, which is rarely reported or considered in study designs or reviews but could be a crucial step to generate reliable data and conclusions.

## 5. Conclusions

Our results suggest that mindfulness-related interventions promote the dampening of BOLD activity in the amygdala in OCD patients [52] and parasympathetic activity, increased vagal tone and PTSD symptom improvements [53,54,55]. However, very limited research was highlighted by our systematic review, and peculiar methodological limitations of the existing studies should be taken into account. Even the results from underpowered and poorly designed RCTs may be overrated because their design granted them unfounded credibility. In addition, inaccurate conclusions derived from these trials may guide clinical practice as clinicians’ decisions may be conditioned by the fact that an RCT design was used [80]. Thus, for contemplative practices research, it is highly recommended to include, and report, an a-priori power analysis with appropriate sample size identification (see, for example, Schmidt et al. [81]).

The results of our systematic review and the documented positive effects of meditation on mental and physical health [82,83] promote contemplative practices as health-related interventions. Science is now interfacing with insights derived from historical and often ancient contemplative practices. The accumulated knowledge suggests that meditative practices not only lead to a different perspective of reality that fosters a connectedness with others expressed through feelings of compassion, but also may have positive influences on health.

## Figures and Tables

**Table 1:** PICOS.

Parameter	Inclusion Criteria	Exclusion Criteria
Population	PTSD and OCD patients. Expert or naïve for mindfulness or compassion techniques	Underaged (<18 years) and/or older (>65 years) subjects
Intervention	Any protocol of mindfulness-related or compassion-related intervention	Slow breathing techniques not related to mindfulness or compassion interventions
Comparison	Comparison of mindfulness-related or compassion-related interventions with control groups (other interventions, no intervention)	
Outcomes	Physiological parameters associated with cardio-respiratory system or central nervous system (i.e., EEG, fMRI, HRV, RSA, and cardio-respiratory synchronization), measured with a psychological/behavioral variable (investigated through a psychometric quantitative tool)	Physiological parameters that are not relevant for the review, or only a psychological/behavioral parameter alone has been investigated
Study design	Within subjects, cross sectional, RCTs, longitudinal, pre-post	A lack of rigor in the description of the methodology (e.g., age, gender, how subjects were recruited, treatments and procedures used that are clearly stated, protocol duration, measured outcomes) and of the experimental set-up (e.g., tools and setups used to measure outcomes), so that replicability is difficult. Case reports.

**Table 2:** Search strategy of the study.

Database	Query	Research in	Items Found
PubMed	1. “mindfulness” OR “MBSR” OR “compassion” OR “self-compassion”	Title/abstract	78,556
	2. “Cardio respiratory coherence” OR “Cardio-respiratory coherence” OR “Cardiorespiratory coherence” OR “Cardiorespiratory coupling” OR “Cardio respiratory coupling” OR “Cardio-respiratory coupling” OR “Cardiorespiratory interaction” OR “Cardio respiratory interaction” OR “Cardio-respiratory interaction” OR “Cardiorespiratory synchronization” OR “Cardio respiratory synchronization” OR “Cardio-respiratory synchronization” OR “Electroencephalogram” OR “EEG” OR “Functional connectivity” OR “Heart rate variability” OR “HRV” OR “Magnetic resonance imaging” OR “MRI” OR “Respiratory Sinus Arrhythmia” OR “RSA”	Title/abstract	725,931
	3. “Post-traumatic stress disorder” OR “PTSD” OR “obsessive-compulsive disorder” OR “OCD”	Title/abstract	107,609
	4. Combine #1 AND #2 AND #3		33
	5. Limit to “Humans”		23
	6. Limit to (English)		23
Scopus	1. “mindfulness” OR “MBSR” OR “compassion” OR “self-compassion”	Title/abstract/Keywords	14,203
	2. “Cardio respiratory coherence” OR “Cardio-respiratory coherence” OR “Cardiorespiratory coherence ”OR “Cardiorespiratory coupling” OR “Cardio respiratory coupling” OR “Cardio-respiratory coupling” OR “Cardiorespiratory interaction” OR “Cardio respiratory interaction” OR “Cardio-respiratory interaction” OR “Cardiorespiratory synchronization” OR “Cardio respiratory synchronization” OR “Cardio-respiratory synchronization” OR “Electroencephalogram” OR “EEG” OR “Functional connectivity” OR “Heart rate variability” OR “HRV” OR “Magnetic resonance imaging” OR “MRI” OR “Respiratory Sinus Arrhythmia” OR “RSA”	Title/Abstract/Keywords	1,232,687
	3. “Post-traumatic stress disorder” OR “PTSD” OR “obsessive-compulsive disorder” OR “OCD”		20,114
	4. Combine #1 AND #2 AND #3		42
	5. Limit to “Humans”		37
	6. Limit to (English)		35

**Table 3:** Included studies.

Study	Study Design	Mindfulness- or Compassion-Related Intervention Group	Control Group	Mean Age (Standard Deviation) [Control Group]	Mindfulness- or Compassion-Related Intervention	Mindfulness- or Compassion-Related Intervention Details	Comparison Intervention(s)	Comparison Intervention(s) Details
Simon et al., 2014	Cross-sectional	21 unmedicated OCD patients (13 females)	21 healthy controls (13 females)	33.1 (10.8) [33.1 (10.1)]	Distracting bar orientation task		Self-referential evaluation task	
Bhatnagar et al., 2013	Cohort	8 PTSD patients (1 female)	No control group	59.5 (range, 42–71 years)	MBSR	8 weeks	No comparison intervention	
Kang et al., 2018	Cohort	29 PTSD patients (6 females)	No control group	59.0 (12.8)	Transcendental Meditation (TM)	8 weeks	No comparison intervention	
King et al., 2016	Pre-Post	14 PTSD patients (0 females)	9 PTSD patients (0 females)	32.43 (7.54) [31.37 (10.14)]	Mindfulness-Based Exposure Therapy (MBET)	16 weeks	Present-Centered Group Therapy (PCGT)	16 weeks
Williams et al., 2020	RCT	80 PTSD patients (females n° not reported)	2 groups of 80 PTSD patients (females n° not reported)	Not reported	Mindfulness Meditation (MM)	8 weeks	Hypnosis, Education condition	8 weeks
Wahbeh et al., 2016	RCT	27 PTSD patients (2 females)	3 groups of 25 PTSD patients (4 females)	53.3 (12.6) [1. 52.2 (12.5) 2. 50.0 (12.8) 3. 53.0 (11.8)]	Mindfulness Meditation (MM)	6 weeks	MM and Slow breathing (SB), SB, Sitting quietly (SQ)	6 weeks

**Table 4:** Outcomes.

Study	Cardio-Respiratory System	Central Nervous System	Psychological/Behavioral Outcome	Statistical Analyses	Power Analysis
Simon et al., 2014	Not investigated	Dampening of BOLD amygdala hyperactivity	Only OCD-related and aversive stimuli were rated less unpleasant during distraction	Repeated measures ANOVA with a Greenhouse–Geisser correction when sphericity was violated	Not reported
Bhatnagar et al., 2013	Increased pNN50 measure of HRV	Not investigated	PTSD symptoms had decreased from baseline by an overall CAPS score of 14.8 points	Not reported	Not reported
Kang et al., 2018	Not investigated	Increased low-frequency bands (1–7 Hz) of EEG spectral power at post-treatment and follow-up and only during meditation states	Reductions in PTSD symptoms, experiential avoidance, and depressive and somatic symptoms, as well as increases in measures of mindfulness and quality of life	Repeated measures ANOVA with a Hyunh-Feldt correction when sphericity was violated	Not reported
King et al., 2016	Not investigated	Increased DMN rsFC with DLPFC and dorsal ACC regions following MBET	Reduction in PTSD symptoms; PCC-DLPFC correlated with improvement in PTSD avoidant and hyperarousal symptoms	Paired-sample t-test comparisons	Not reported
Williams et al., 2020	Not investigated	EEG pre- and post- treatment (protocol only)	Mindfulness, Self-compassion, Pain catastrophizing and Pain acceptance measures (protocol only)	ANOVA (protocol only)	N = 240 to have at least 80% power, even at the largest standard deviation when using ANOVA
Wahbeh et al., 2016	No change in HR and HRV within and between groups	Lower awakening cortisol within MM group	Subjective hyperarousal symptoms improvement within-group (but not between groups) for MM, MM+SB, and SQ; Intrusive thoughts decreased in MM compared with MM + SB and SB	Paired t-test; ANCOVA	N = 25 in each group, for the main effects of ANCOVA the adjusted effect size (f) is 0.46, which yields power of >0.99

## Data Availability

No new data were created in this study. Data sharing is not applicable to this article.

## Supplementary Materials

The following are available online at https://www.mdpi.com/article/10.3390/ijerph182211778/s1, Table S1: JBI critical appraisal checklist for cross-sectional studies; Table S2: JBI critical appraisal checklist for pre-post studies, Table S3: JBI critical appraisal checklist for cohort studies; Table S4: JBI critical appraisal checklist for RCT studies.
