# Supplementary material for: A Systematic Review of a Polyvagal Perspective on Embodied Contemplative Practices as Promoters of Cardiorespiratory Coupling and Traumatic Stress Recovery for PTSD and OCD: Research Methodologies and State of the Art
Source: Int J Environ Res Public Health. 2021 Nov 10;18(22):11778. doi: 10.3390/ijerph182211778 (PMC8619958; doi:10.3390/ijerph182211778)
Supplement: Supplementary file 1 [file ijerph-18-11778-s001.zip › ijerph-1433529-supplementary.pdf]

Supplementary Materials

Supplementary Table S1. JBI critical appraisal checklist for cross-sectional studies

	**Simon *et al.* 2014**			
	Yes	No	Unclear	NA
1. Were the criteria for inclusion in the sample clearly defined?	X			
2. Were the study subjects and the setting described in detail?	X			
3. Was the exposure measured in a valid and reliable way?			X	
4. Were objective, standard criteria used for measurement of the condition?	X			
5. Were confounding factors identified?			X	
6. Were strategies to deal with confounding factors stated?			X	
7. Were the outcomes measured in a valid and reliable way?	X			
8. Was appropriate statistical analysis used?	X			

Supplementary Table S2. JBI critical appraisal checklist for pre-post studies

	**King *et al.* 2016**			
	Yes	No	Unclear	NA
1. Is it clear in the study what is the 'cause' and what is the 'effect' (i.e. there is no confusion about which variable comes first)?	X			
2. Were the participants included in any comparisons similar?	X			
3. Were the participants included in any comparisons receiving similar treatment/care, other than the exposure or intervention of interest?	X			
4. Was there a control group?	X			
5. Were there multiple measurements of the outcome both pre and post the intervention/exposure?	X			
6. Was follow up complete and if not, were differences between groups in terms of their follow up adequately described and analyzed?		X		
7. Were the outcomes of participants included in any comparisons measured in the same way?	X			
8. Were outcomes measured in a reliable way?	X			
9. Was appropriate statistical analysis used?	X			

Supplementary Table S3. JBI critical appraisal checklist for cohort studies

	**Bhatnagar *et al.* 2013**				**Kang *et al.* 2018**			
	Yes	No	Unclear	NA	Yes	No	Unclear	NA
1. Were the two groups similar and recruited from the same population?				X				X
2. Were the exposures measured similarly to assign people to both exposed and unexposed groups?				X				X
3. Was the exposure measured in a valid and reliable way?	X				X			
4. Were confounding factors identified?		X					X	
5. Were strategies to deal with confounding factors stated?		X					X	
6. Were the groups/participants free of the outcome at the start of the study (or at the moment of exposure)?			X		X			
7. Were the outcomes measured in a valid and reliable way?	X				X			
8. Was the follow up time reported and sufficient to be long enough for outcomes to occur?	X				X			
9. Was follow up complete, and if not, were the reasons to loss to follow up described and explored?			X		X			
10. Were strategies to address incomplete follow up utilized?		X					X	
11. Was appropriate statistical analysis used?			X		X			

Supplementary Table S4. JBI critical appraisal checklist for RCT studies

	Wahbeh et al. 2016				Williams et al. 2020			
	Yes	No	Unclear	NA	Yes	No	Unclear	NA
1. Was true randomization used for assignment of participants to treatment groups?	X				X			
2. Was allocation to treatment groups concealed?	X				X			
3. Were treatment groups similar at the baseline?	X							X
4. Were participants blind to treatment assignment?	X				X			
5. Were those delivering treatment blind to treatment assignment?		X				X		
6. Were outcomes assessors blind to treatment assignment?	X				X			
7. Were treatment groups treated identically other than the intervention of interest?	X				X			
8. Was follow up complete and if not, were differences between groups in terms of their follow up adequately described and analyzed?		X			X			
9. Were participants analyzed in the groups to which they were randomized?	X				X			
10. Were outcomes measured in the same way for treatment groups?	X				X			
11. Were outcomes measured in a reliable way?	X				X			
12. Was appropriate statistical analysis used?	X				X			
13. Was the trial design appropriate, and any deviations from the standard RCT design (individual randomization, parallel groups) accounted for in the conduct and analysis of the trial?	X				X			
